# The QICKD study protocol: a cluster randomised trial to compare quality improvement interventions to lower systolic BP in chronic kidney disease (CKD) in primary care

**DOI:** 10.1186/1748-5908-4-39

**Published:** 2009-07-14

**Authors:** Simon de Lusignan, Hugh Gallagher, Tom Chan, Nicki Thomas, Jeremy van Vlymen, Michael Nation, Neerja Jain, Aumran Tahir, Elizabeth du Bois, Iain Crinson, Nigel Hague, Fiona Reid, Kevin Harris

**Affiliations:** 1 Division of Community Health Sciences, St George's – University of London, London, SW17 0RE, UK; 2 SW Thames Institute for Renal Research, St Helier Hospital, Carshalton, Surrey, SM5 1AA, UK; 3 Department of Public Health Primary Care and Food Policy, City Community and Health Sciences, City University, 20, Bartholomew Close, London, EC1A 7QN, UK; 4 Kidney Research UK, Kings Chambers, Priestgate, Peterborough, PE1 1FG, UK; 5 Public Health Department, Wandsworth PCT, Wimbledon Bridge House (3rd Floor), 1, Hartfield Road, London, SW19 3RU, UK; 6 University Hospitals of Leicester, John Walls Renal Unit, Leicester General Hospital, Leicester, LE5 4PW, UK

## Abstract

**Background:**

Chronic kidney disease (CKD) is a relatively newly recognised but common long-term condition affecting 5 to 10% of the population. Effective management of CKD, with emphasis on strict blood pressure (BP) control, reduces cardiovascular risk and slows the progression of CKD. There is currently an unprecedented rise in referral to specialist renal services, which are often located in tertiary centres, inconvenient for patients, and wasteful of resources. National and international CKD guidelines include quality targets for primary care. However, there have been no rigorous evaluations of strategies to implement these guidelines. This study aims to test whether quality improvement interventions improve primary care management of elevated BP in CKD, reduce cardiovascular risk, and slow renal disease progression

**Design:**

Cluster randomised controlled trial (CRT)

**Methods:**

This three-armed CRT compares two well-established quality improvement interventions with usual practice. The two interventions comprise: provision of clinical practice guidelines with prompts and audit-based education.

The study population will be all individuals with CKD from general practices in eight localities across England. Randomisation will take place at the level of the general practices. The intended sample (three arms of 25 practices) powers the study to detect a 3 mmHg difference in systolic BP between the different quality improvement interventions. An additional 10 practices per arm will receive a questionnaire to measure any change in confidence in managing CKD. Follow up will take place over two years. Outcomes will be measured using anonymised routinely collected data extracted from practice computer systems. Our primary outcome measure will be reduction of systolic BP in people with CKD and hypertension at two years. Secondary outcomes will include biomedical outcomes and markers of quality, including practitioner confidence in managing CKD.

A small group of practices (n = 4) will take part in an in-depth process evaluation. We will use time series data to examine the natural history of CKD in the community. Finally, we will conduct an economic evaluation based on a comparison of the cost effectiveness of each intervention.

**Clinical Trials Registration:**

ISRCTN56023731. ClinicalTrials.gov identifier.

## Background

Chronic kidney disease (CKD) is a common long-term condition, affecting 5 to 10% of the population. CKD is an independent risk factor for cardiovascular disease, established renal failure (ERF) and all cause mortality [1-3]. Patients with CKD are far more likely to die prematurely from cardiovascular disease than progress to ERF requiring dialysis or transplantation. The presence of proteinuria confers additional cardiovascular risk.

CKD is classified into five stages based upon a measurement of kidney function and the estimated glomerular filtration rate (eGFR) determines the class of CKD for the more severe stages (Stage three to five). Stage one and two are the mildest of the five stages of CKD and require evidence of kidney damage, usually the presence of proteinuria, to confirm the diagnosis. Stages three to five CKD can be diagnosed by eGFR alone; and stage three is now often split into stages 3a and 3b, as there are far higher rates of cardiovascular co-morbidity in stage 3b disease. People with cardiovascular co-morbidities especially hypertension and diabetes; cardiovascular risk factors, particularly raised systolic blood pressure (BP); and more specific renovascular risk factors: proteinuria and anaemia are at increased risk.

There is a broad and evidence-informed consensus that lowering BP is of central importance, both to slow the progression of CKD and reduce cardiovascular risk. Lowering of BP using angiotensin modulating anti-hypertensives, angiotensin converting enzyme inhibitors (ACEI) and angiotensin (II) receptor blockers (ARB) appears to have additive renal-protective benefits [4]. Strict management of BP, cardiovascular and specific renovascular risk should be feasible in primary care. Guidelines on the management of CKD have recently been published by the National Institute for Health and Clinical Excellence (NICE) [4]. In the absence of proteinuria, the threshold for intervention is a BP of ≥ 140/90 mmHg is recommended, with a target systolic BP of between 130 and 139 mmHg. In diabetes and where significant proteinuria is present, the respective values are 130/80 mmHg with a systolic target of between 120 and 129 mmHg. However these targets frequently remain unmet. Studies have demonstrated a need to improve both information and training available to practitioners with the aim of enabling them to improve the quality of care currently provided [5].

There is limited knowledge and experience of managing this condition in primary care, and while CKD has been included as one of the financially incentivised chronic disease management targets for general practice – the 'Quality and Outcomes Framework' (QOF) it is the only QOF indicator to be accompanied by a 'Frequently Asked Questions' document – requested by the British Medical Association as a condition for the inclusion of this indicator in the QOF indicator set [6]. Feedback to the investigators has been that practitioners lack confidence in the management of this condition, especially implementing the BP targets in elderly patients (who are at higher risk of CKD and its sequelae).

There are further problems with the QOF. The use of routinely collected clinical data for purposes other than clinical care may distort data recording [7]. Practitioners feel reluctant to include a patient with incomplete data on a QOF disease register as this might affect their income. Regardless, the prevalence of CKD reported through the QOF to the NHS Information Centre for 2006/7 [8] is less than half that reported in the epidemiological studies quoted in this introduction. There is *de facto *a quality gap as those people with CKD not on the disease register will not be recalled for BP and other checks.

Finally, the new NICE guidance looks at CKD at a point in time [4]. Management is largely determined by the eGFR over a three-month period, BP control and the presence or absence of proteinuria. Although there is a heuristic for a rate of decline that would trigger referral, there is dissonance between this heuristic and clinical practice in primary care. Many elderly people with CKD, even more advanced stage four disease, appear to be stable and the NICE along with previous guidance may be over aggressive for this group of patients; this may be part of the reason why clinicians are not implementing recommended BP targets [9,10]. Further research is needed to understand the natural history of the disease and whether rate of decline would be a more appropriate primary variable to detect people at risk.

### The quality of general practice computer data

UK general practice is almost universally computerised and has some of the most advanced general practice computing [11,12]; providing a rationale for the use of routinely collected data to measure the impact of the quality improvement interventions being developed and tested in this programme of research. Six factors contribute to the high quality of general practice computer data: we have an accurate denominator [13]; prescribing records are largely complete; electronic connections to laboratories mean that pathology data are complete; the QOF has improved data quality in CKD and its cardiovascular co-morbidities including diabetes; an electronic referral system has improved data quality; and the NHS has sponsored the development of a tool called MIQUEST (Morbidity Information Query and Export Syntax) to extract anonymised data – a tool we have over 10 years experience of using [14,15].

Optimal management of CKD in primary care is currently limited by a lack of knowledge about how to increase adherence to guidelines for best practice [16]. There is no single perfect quality improvement strategy to use in primary care [17]. The most commonly used strategy is dissemination of clinical practice guidelines with prompts [18]. This usually involves distribution of paper guidance and reminders with internet resources providing additional information and support. More expensive and complex interventions have been widely used, including audit-based education (ABE) where practitioners compare their own practice's adherence to guidance with that of peer practices [19,20]. Our experience from observational work has been that ABE is more effective in its second year [21]; a similar pattern is seen with using feedback to improve data quality [22].

## Methods

### Study aims and objectives

This study aims to improve the quality of CKD management in primary care with the emphasis on strict control of systolic BP to reduce cardiovascular risk and slow renal progression.

### Objectives

1. To lower the BP of hypertensive individuals with CKD to an agreed target.

2. To measure the impact of the quality improvement interventions on the recording and control of renovascular risk factors, including proteinuria; and cardiovascular co-morbidities, including diabetes mellitus.

3. To evaluate the quality improvement interventions and measure their impact on other markers of quality, including practitioner confidence.

4. To establish a cost model for each quality improvement intervention.

5. To characterise the natural history of CKD. We wish to compare those who have progressive (as defined by a yearly decrease in eGFR of >5 ml/min/1.73 m^2 ^in one year or >10 ml/min/1.73 m^2 ^in 5 years) [4], compared with non-progressive renal disease; comparing demographics, co-morbidities (including diabetes), and biomedical variables.

6. To develop improved primary care guidelines for management of CKD and measure adherence to this guidance; with an emphasis on comparing progressive, with non-progressive CKD.

### Study design

#### Study design overview

We plan to conduct a two-year, three-arm cluster randomised trial. We are carrying out a cluster randomised trial because we feel that quality improvement is often adopted at the level of the practice. A trial of individual patients would be much more difficult because it may be impossible to stop contamination between general practitioners (GPs) and other health professionals working in the same practice; GPs may see successive patients from different arms of the trial; and communication between patients randomised to different arms of the trial might also bias results.

**Figure 1:**
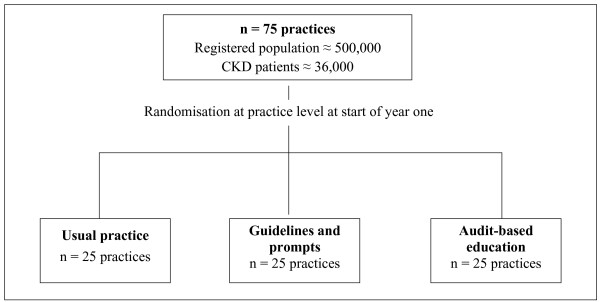
The core study sample: a three-arm cluster randomised trial comparing Usual practice with Guidelines and Prompts (GaP) and Audit-based Education (ABE).

**Figure 2:**
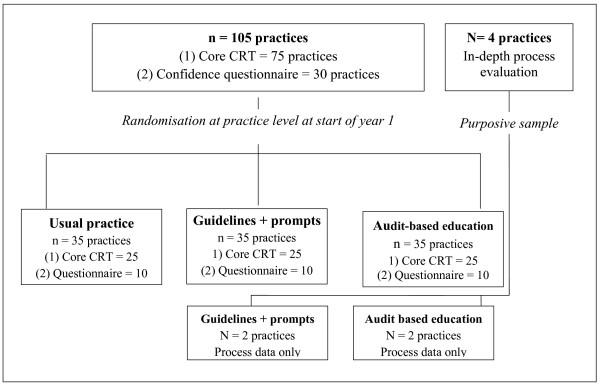
**The greater study contains the core CRT with 25 practices in each arm.** In addition there are 10 confidence questionnaire practices per arm and two in-depth process analysis practices in each of the active study arms.

The study has two components: a core cluster randomised trial (CRT) of 75 practices, and a parallel process evaluation and measure of how GP confidence changes over time. The core study is a three-arm CRT of 75 practices. These 75 practices are randomised into three arms of 25 practices comparing usual practice, guidelines and prompts (GaP), and ABE. This sample size is needed to show a difference of 3 mmHg in systolic BP (Figure 1). There is also a parallel study that contains additional practices: four practices form our in-depth process evaluation practices, and two testing each active intervention. Additionally, 10 practices in each arm of the study will complete a confidence questionnaire to assess if/how practitioner confidence changes in the different arms of the study (Figure 2).

However, the parallel study (Figure 2) contains two other elements:

1. Four in-depth process evaluation practices: These practices will take part in our diagnostic analysis process at the start of the study proper (*i.e*., does the intervention meet their perceived needs, and does it address barriers to quality improvement). They will validate our questionnaire to assess confidence and, during the study proper, report on the intervention exposure (*i.e*., to what extent the intended recipients are exposed to the interventions); and programme fidelity (*i.e*., whether the quality improvement intervention is delivered as planned). Two practices will give in-depth feedback about the GaP intervention and two about ABE. We will use focus groups run in each practice as our principal method of data collection; however we also plan a mid-study workshop of all the in-depth process evaluation practices. Our sample will include at least one practice from the north and from the south; we intend to recruit inner city, suburban, and county town practices; we want to see the four major brands of general practice computer systems represented across the practices so that we can also test our queries and data extracts.

2. An additional 10 practices in each arm will complete a confidence questionnaire: We will recruit 10 additional practices in each arm that will participate in the study but also complete a questionnaire about their confidence in the management of CKD. We are primarily doing this to assess if any of the interventions have a greater effect on confidence. We are sending this questionnaire to a separate group of practices because completing the questionnaire may be an intervention in its own right, possibly as great as GaP. We will be able to compare questionnaire and non-questionnaire practices in each arm at the end of the study.

#### Participants

The participants are GPs located in practices (our clusters) across England. We aim to recruit a nationally representative sample of practices from: in and around London – especially inner city and suburban southwest London; urban and rural Surrey and Sussex; Leicester city and surrounding areas; Birmingham inner city and suburban; and Cambridge. The locality structure is pragmatic because groups of practices need to come together for the ABE workshops. An inclusion criteria for a locality is that their local renal unit would support the workshop within their locality and review the GaP to minimise any conflict with local policy.

The primary research participants are GPs involved in the study who will receive the quality improvement interventions listed below. The interventions will be implemented at the practice (cluster) rather than the individual level. The study subjects (who may be regarded as secondary participants) will be all individuals with CKD within the study practices. CKD will be defined using the internationally accepted National Kidney Foundation (NKF) definition [23] using two measures of eGFR of less than 60 ml/min/1.73 m^2 ^at least three months apart. However, we will also explore the effects of including people with a single recording of eGFR.

The participants do not receive any financial incentives to participate, though they do receive financial compensation for the time actually spent attending study activities. These will vary according to the arm of the study they are allocated to.

### Inclusion and exclusion criteria

#### Inclusion criteria

1. Localities require the local renal unit to share local guidance and support our interventions.

2. Primary care organisation approval for the research to be conducted in their locality.

3. Practices who provide written consent to participate.

4. Agreement to participate in whichever arm of the study they are randomly allocated.

5. Practice has had the same computer system for the last five years and has no plans to change it, and will allow access to check data quality.

6. Practice has electronic laboratory links for three years or more.

#### Exclusion criteria

1. Practices in whom the computing system has changed over the last five years.

2. Practices lacking an appropriate computer system from which data can be extracted.

3. Practices in which referral data (from primary care to secondary care) is not available.

4. Practices planning to move computer system in the next two years.

#### Recruitment

Dedicated members of the study team (NT and NJ) liaise with and recruit eligible practices from the study's 'localities' who meet with the above inclusion criteria. The primary care research networks, funded by the National Institute for Health Research (NIHR) have actively supported the recruitment for the study in all of our target areas since the project was added to the NIHR portfolio of research projects. Recruitment has also been carried out by writing to practices associated with teaching networks in southwest London, Surrey and Sussex (SdeL). There has been word-of-mouth recruitment from members of the project team, and snowball recruitment from practice to practice.

#### Consent

Practices will be asked to consent as a unit, with all GPs being willing to participate. One or more persons will sign the consent form as authorised by the practice. This may vary from all GPs to one GP being authorised to consent on behalf of the practice. No direct consent is taken from patients, however a waiting room poster is provided as well as a lay summary of the project in leaflet form.

### Interventions

#### The interventions in the study

Two interventions are being compared to usual practice: GaP and ABE. The interventions are designed to target the cluster (*i.e*., individual general practices). Where we send GaP or questionnaires we send them to individual named clinicians. Where a practice is invited to attend an ABE workshop all members may attend; however, our experience is that one or more practice members attend on behalf of the others; we try to compensate for this by providing learning resources for them to take back to their practices. However, although we send some material to individuals, the intervention is focused at the level of the practice.

##### Usual practice

These practices will be allocated to this arm at randomisation (n = 35 practices – 25 in the core CRT and 10 in the questionnaire group). Once assigned to this arm, a minimum of contacts will be made of these practices other than for data collection.

##### Distribution of clinical practice guidelines with prompts (GaP)

This is an established, low cost method of quality improvement [17]. It will provide a benchmark with which the effectiveness of the other quality improvement intervention can be compared. We will develop a consensus between the study team, our expert advisory group, and external peer reviewers, and produce appropriate guidance for the management of CKD in primary care. This guidance will be distributed to practices within this arm of the CRT (n = 25 practices plus 10 questionnaire practices) with six monthly updates and reminders. The guidance will be customised to fit with local practice and reflect guidance in that area. In addition practices will have access to a supportive website with information about CKD, frequently asked questions, and tools to improve CKD management.

The GaP documentation will typically be up to four sides of A4 paper stock, published in a glossy professionally printed form. It may be accompanied by local guidance or national brief guidance in the first intervention. We plan to distribute the NICE 'Quick Reference Guide' to managing CKD [24] as part of the second-year intervention.

##### Audit based-education (ABE)

In this arm, practices (n = 25 practices plus 10 questionnaire practices) will have a representative attend workshops. These practices will also have access to clinical practice guidelines provided to the second arm of the study. However, in addition, practices will receive three sets of detailed comparative feedback about their quality of CKD management at approximately nine-month intervals, and we will facilitate lists of patients needing interventions (local queries) being created within the practice. This comparative feedback about adherence to guidelines will be based on anonymised data collected from their general practice computer system prior to the ABE workshop.

The study will use an ABE model for quality improvement developed by the primary care data quality project that has been used in a variety of clinical contexts [19]. This involves feedback given in a workshop setting with at least one GP and one nurse or practice manager from each practice present. The workshops will be in two parts: the first will be facilitated by a GP familiar with the data, ideally from the locality, but if not, available from study team, and a local renal specialist in attendance to provide expert advice and information about local practice. The first part will be a presentation of the comparative adherence to evidence-based guidance for the management of CKD by the different practices present led by the GP. This section will highlight variation in the quality of care in a non-judgemental context. The second part of the meeting will be case studies, facilitated by the local consultant, which small groups will work through to explore dilemmas in management and how to overcome them.

The workshops are timetabled for two and a half hours of activity with additional break time to allow informal contact. Practices are expected to bring along at least one GP and one or two other members of the practice team: their practice manager and a nurse involved in cardiovascular risk assessment or diabetes within the practice.

Delegates are asked to fill in a feedback form, of the standard type used to evaluate educational meetings, on the usefulness and appropriateness of the content and the educational methods used. There is also the opportunity to provide informal feedback. This feedback, along with a narrative from the three members of the study team who participate in these workshops (it is expected there will be at least three) will be fed back into the design of subsequent feedback. Semi-structured interviews – reviewing the appropriateness of the level; the content and the delivery are being held in person or by telephone with all members of the study team who had attended or participated in the first round of workshops.

#### The content of the interventions

The content and focus of the GaP arms of the study will be the same as in the ABE arm. The areas and learning objectives for each year have been set; however, the specific details will depend on the national guidance available at the time. Currently, we are basing our year one criteria and standards on the NICE guidance released in September 2008 [4].

#### Year one

During the first year, the clinical focus will be on understanding any gap between the 'true' prevalence revealed by the audit and the 'QOF prevalence' the practice reported to the NHS Information Centre, which is publicly available information [8]. We expect our audit to identify approximately double the number of people with CKD than included in the practice QOF disease register. In addition, this year will look at proteinuria recording, control of BP and use of appropriate therapy: angiotensin modulating drugs, appendix 1.

#### Year two

The second year's clinical focus will be on the management of co-morbidities, especially diabetes. Strict control of cardiovascular risk factors in patients with CKD and Cardiovascular System (CVS) risk is important. We also look at control of BP in diabetes. People with diabetes and CKD need stricter BP control, especially if they have microalbuminuria; diabetics are also one of the most likely groups to go on to require renal replacement therapy, appendix 2.

### Outcome measures

Our primary care outcome measure is change in systolic BP in people with hypertension and stage three to five CKD. We have secondary outcome measured in the following categories:

1. What happened: Clinical outcomes and change in practitioner confidence.

2. Why change happened: Diagnostic analysis plus process evaluation.

3. What it cost: Economic evaluation.

4. Unexpected consequences.

### Primary outcome measure

The primary outcome measure is the reduction of systolic BP in hypertensive people with Stage three to five CKD towards the current national target [4]. [Hypertension is defined as above >140 mmHg in low-risk patients and >130 mmHg in high-risk patients. High-risk patients are people with CKD plus significant proteinuria (ACR ≥ 70 mg/mmol; or equivalent) or with CKD and diabetes.

We plan to measure the effect of the intervention across the same cohort, though we recognise that it will have less effect on people in stage four and five CKD, as these people are largely managed by specialists. However, as they represent a small percentage of the people with stage three to five disease (<5%) [5], we think this is unlikely to significantly distort our results. We will also explore the effect of the intervention on people older than 75 years.

### Secondary outcome measures

In addition to measuring the effect of the various quality interventions upon systolic BP, will study a number of secondary outcomes:

### Clinical and laboratory markers

1. Case definition using eGFR: We will define cases using the internationally accepted definition used by NKF Kidney Disease Outcomes Quality Initiative (KDOQI) [23], the same definition is used by NICE [4]. We will identify cases recorded since the standardisation of creatinine recording in 2006. However, we will also undertake a sensitivity analysis, including how prevalence changes when the date or number of readings are changed.

2. BP: We will measure the proportion of people in each arm with hypertension and CKD who achieve at least a ≥ 5 mmHg reduction in systolic BP. The reduction of mean systolic BP (the primary outcome measures) could be distorted in a number of ways.

3. Recording and management of key co-morbidities: diabetes and its complications; ischaemic heart disease; heart failure; obstruction/lower urinary tract symptoms.

4. Recording and management of other cardiovascular risk factors: smoking status; lipid management; proteinuria; anaemia; glycated haemoglobin and microalbuminuria in people with diabetes.

5. Serial measures of serum creatinine concentration and eGFR: to explore natural history and look for cases of accelerated decline (defined as a reduction of eGFR of >5 ml/min/1.73 m^2 ^in one year; or >10 ml/min/1. 73 m^2 ^in five years) [4].

6. Recording of death and cause of death: Although this is incompletely recorded, we will attempt to capture any recording as we expect mortality among hypertensive people over the period of the study. There may be a higher mortality among those who are in the control than intervention arms.

7. Avoiding harm: We wish to monitor whether BP reduction is associated with an increased number of falls particularly in older people. Most people with CKD are elderly and at potential risk for falls. Notwithstanding the results of recent systematic reviews that failed to show an association between falls and anti-hypertensive medication [25,26], this remains a genuine concern to some practitioners, and one that we propose to examine. A falls dataset will be devised and integrated into the renal dataset. We will investigate the relationship with use of ACE inhibitors and angiotensin II receptor blockers and systolic BP below 120 in CKD.

8. Medicines management

8. a. Use of drugs/therapy that affect renal function (for example non-steroidal anti-inflammatory drugs)

8. b. Use of ACEI and angiotensin II receptor blockers to control hypertension

8. c. Recording of medicines possession ratio based on days prescribed therapy as an index of concordance with anti-hypertensive therapy.

The details of our dataset are shown in appendix 3.

### Diagnostic analysis and process evaluation, including confidence and end of project questionnaires

1. Practitioner confidence to be measured at t = 0, t = 18 months using a questionnaire that assesses confidence.

2. Feedback from focus groups held prior to round one (diagnostic analysis).

3. Feedback from focus groups held mid-study and at the end of the study.

4. End of study questionnaire and workshops.

### Economic evaluation

We know the economic impact of implementing guidance in place prior to the publication of NICE guidance in September 2008 for the primary care management of CKD [27]. We will update the model used by Klebe *et al*. to reflect the restriction of investigations for renal bone disease in current guidance [4] compared with those advocated in previous guidance [28]. We will then compare the projected investigation cost with the true costs as represented in the routinely collected data.

### Unexpected consequences

We wish to capture any unintended consequences through our process evaluation arm, especially via the open questions in each year of the study (appendices 1 and 2). Many implementations of IT-based change have unintended consequences [29]. Specifically, we will explore with process improvement practices any issues about calling in or recalling patients, and any adverse reactions to therapy or interactions; we will also look at the rates of collection of prescriptions for ACEI and ARB as a proxy for medicine possession ratio. Quality improvement strategies based on open sharing of data may also have unintended consequences [30-32]; though in this study our data sharing is largely within the peer group rather than with the public.

### Data quality assurance

The study has been designed and will be reported in accordance with the CONSORT (Consolidated Statement of Reporting Trials) and its extension to cluster randomised trials [33]. Data will be controlled in accordance with data protection legislation, institutional protocols of St. George's University of London, and NHS policies for research and information governance for ensuring patient confidentiality [34]. Data will be analysed in SPSS (Statistical Package for Social Sciences) version 15 using an intention to treat approach.

### Biomedical data

These data will be extracted from general practice computer systems using the department of health sponsored data extraction system MIQUEST. MIQUEST has been developed by the NHS and is used in the national data quality programme at PRIMIS (Primary Care Information Services) [35]. This application allows identical searches on different brands of general practice computer systems. MIQUEST, when written in its 'remote' mode, extracts pseudo-anonymised clinical data. In its 'local' mode, it allows the extraction of patient identifiable data, such as postcodes for mapping onto multiple deprivation index, and for case-finding within the practice.

Routinely collected general practice computer data are complex and require significant processing and interpretation in order to obtain meaningful information [36]. The research team has considerable experience and has developed a published method [37]. The research data will be completely traceable due to the development of a sophisticated meta-data schema [38,29]. Our extraction technique includes thorough piloting and planning, and data processing with quality controls at each stage. All variables are examined for their distribution, and cleaned appropriately. Where possible, we use therapy and/or pathology tests to triangulate diagnostic and symptom codes.

An issue with routine data is that they are incomplete, and in contrast with other trial data are not systematically recorded at regular intervals. However, we expect to have relatively complete data on people with cardiovascular co-morbidity for the last five years (since the 2004 new contract for general practice) and hopefully longer. The quality of UK primary care data continues to increase, and there is a growing amount of published research that is based on routinely collected data – especially from countries with registration based primary care [14].

We have an agreement with CKD researchers in Galway, Ireland, who have experience of using routinely collected data to research CKD [39,40], that they will independently scrutinise our analysis procedures and generation of results tables.

### Diagnostic analysis and process evaluation

The questionnaire to test practitioner confidence has been developed using a standard questionnaire development method [41]. This questionnaire, developed by GP experts and renal specialists, has been validated through initial testing within the study team, then tested within a south London practitioners group who are not participants in this study. Finally, it was tested within our process evaluation group. The questionnaires are sent to individual health care professionals participating in this study; they are numbered so that reminders can be sent and survey data at the different time points can be inked. Reminders are sent by post. There will also be a final reminder by telephone.

The focus groups are led by members of the study team after receiving training from an experienced qualitative researcher, IC. The focus groups are recorded and transcribed verbatim before IC undertakes more detailed analysis. The analysis will utilise the 'framework' approach developed at the National Centre for Social Research and now a widely used method for analysis within the field of health and social care research [42]. The emergent themes will be discussed with the study team. Focus groups will be continued until thematic saturation is reached.

### Economic evaluation

The Health Foundation is providing expert health economic consultancy to the quality improvement projects. Once our first-round data collection is complete, we will review this with the expert advisors [43].

### Sample size

#### Cluster randomised trial sample size

SK, an experienced medical statistician with specific expertise in cluster randomised trial design [44,45], conducted a sample size calculation taking into account variation between practices. The study is powered to detect a >3 mmHg difference in systolic BP between the groups over the two-year duration of the study. Because of the large number of patients per cluster, the sample size can be estimated using a 'summary statistic' approach whereby each practice provides a single mean BP. Using a sample dataset of 30 practices, we have estimated that the variation between practice means has a standard deviation (SD) of 3.77 mmHg. Assuming that this sample of 30 practices is representative of the study practices in terms of their size and number of CKD patients, a sample size of 25 practices per intervention group will be required to detect a difference of 3 mmHg at the 5% level with a power of 80%.

The intra-cluster correlation coefficient (ICC) is estimated to be approximately 0.03. There are likely to be approximately 500 patients (m) with CKD per practice (based on a disease prevalence of 6.5%). We can use this information to calculate the design effect. The design effect or inflation factor is the extent to which likely correlation with a cluster (in our case an individual practice) increased the sample size required.



A larger difference of clinical importance (*e.g*., 5 mmHg) would require a smaller sample. However, given the population nature of this intervention, we decided to be prudent and power the study for a small difference.

### Questionnaire survey

A sample of 10 practices in three arms should enable us to compare changes in confidence in managing CKD. We expect to recruit practices with a mean practice list size of around 8,000 [46]. The latest workload survey suggested that 62% of GPs work full time [47]. There is approximately one GP per 1,700 patients. The confidence questionnaire adopts a five-point scale.

We estimate that there will be at least two practice nurses per 8,000 patients engaged in assessment of cardiovascular risk including management of CKD. We estimate an average of 10 practitioners per practice are eligible to complete the questionnaire and that we will achieve a >60% response rate, or 180 returned questionnaires.

A pilot study as part of the development of the questionnaire shows that the responses have a mean score of two, and standard deviation of about 1.26. We want to have a power of 0.80, or equivalently, the probability of a Type II error of 0.20, the sample size needed to show a change of 0.5 units in the five-point scale, the smallest individual change meaningful for the study, is 33 practitioners in each arm of the study.

### Stopping rules

Although negative effects are unlikely, any suspected negative effects will be investigated and the study suspended, pending review. The principal safety monitoring activities will be: the observation for falls in people newly started on additional BP lowering drugs; and to identify whether there is any relationship between systolic BP and rate of falls.

### Randomisation

#### Randomisation was conducted in blocks

Practices agree to participate in the study the basis that they will be assigned at random to an arm of the study. We excluded practices who wanted to choose an arm of the study. They are assigned their arm by simple random allocation. Randomisation will be performed with a table of random numbers by JvV; in the order practices complete their consent to participate. He allocates, at random, recruited practices in blocks of nine; accepting that there will be a final block of less than nine.

#### Allocation concealment

The allocation is not shared with those who will be involved in the data analysis. The clinical data collected are identical in all three arms of the study, so there should be no clues within these data as to which arm is which. The allocated arm is recorded in our database of practice details that is kept entirely separately from the pseudonymised table of data used for analysis. Within the analysis table the practices in each of the three arms are identifiable for analysis – but there is no labelling of which specific arm any practice is allocated to. Similarly, patient and practice identifiers are pseudonymised, which again makes it harder for the analysts to identify individual arms.

Ten practices in each arm are labelled as having had the questionnaire. The four in-depth process evaluation practices have a separate series of identification numbers so that they can have their data analysed but excluded from the study.

#### Blinding

The field team are aware of which practices are in which arm, because they must mail or invite participants to the relevant intervention. However, patient and practice details are pseudonymised. All cleaning and processing of data are carried out on the whole database (*i.e*., all three arms) simultaneously. We will do this by only revealing the arm allocation variable at the end of the study. We try to minimise access to signature data that would allow the arms of the study to be differentiated. (*e.g*., if an analyst knew the precise list size of one practice in the study.) However, we only plan to reveal this variable when it is needed for final comparison between arms.

### Statistical analysis

Processed data extracted from GP practices and survey data using questionnaires will be imported onto the SPSS or a compatible software system. The data analysis will be conducted in three stages:

### Univariate and bi-variate analyses

1. We will document the recorded prevalence of CKD, as defined by socio-demographic (*e.g*., age, gender, ethnicity, deprivation scores).

2. We will document the level of confidence of primary care practitioners in the management of CKD stage three to five as defined by age, role, and the characteristics of the GP practices.

3. We will compare the recorded management of CKD Stage 3 – 5 in the participating GP practices with national and local guidelines.

4. We will document the recorded key co-morbidities of CKD stage three to five (*e.g*., diabetes, ischaemic heart disease etc).

5. We will compare the recorded management of key co-morbidities in the participating GP practices with national and local guidelines.

6. We will document the association between management of CKD using BP medication and falls.

### Multivariate techniques

1. Using analysis of variance (ANOVA) models, compare the mean systolic BP of people with CKD stage three to five in the three arms of the study, before and after the interventions – the primary outcome measure of this study

2. Using ANOVA models, compare the confidence level of primary care practitioners in the management of people with CKD Stage three to five in the three arms of the study, before and after the interventions

3. Using multiple regression analyses, explore and quantify relations between independent variables (*e.g*., known demographics and risk factors, such as smoking status, level of cholesterol, obesity, anaemia and alcohol consumption) and dependent variables (*e.g*., CKD stage three to five, and diabetes).

### Longitudinal data analyses

The temporal dimension of the recorded clinical data collected contemporarily offers an opportunity for analyses of the natural history and the disease course of CKD. The data have an advantage of being free from bias from retrospective recall, and allow the follow-up of the full spectrum of the impact of contributory risk factors on and outcomes for people with CKD. A particular interest is the association between management of CKD, the rate of change of eGFR, falls, and the outcomes of CKD.

## Discussion

This study fills a gap in the literature about how to improve the management of CKD in primary care. This gap is worth filling, because interventions that can be administered in primary care should be able to slow the progression of CKD, and consequently reduce cardiovascular co-morbidity and the need for dialysis and transplantation.

The study is a pragmatic approach to quality improvement (QI) in CKD, and is intended to inform practitioners and the commissioners of care about the cost effectiveness of GaP and ABE in this disease area.

The ethical oversight of quality improvement projects remains a subject of much debate [48]. The study does not mandate any new intervention to be given to patients in participating practices, but rather promotes the implementation of best practice. Personalised decisions to treat patients will be made by individual practitioners in partnership with their patients, as now. Indeed, the primary research participants of the study are the participating practitioners rather than they patients they treat. This distinction has been recognised by the ethics committee that approved the study; our view is that studies of this potential size and impact should be part of the ethical approval process. Strictly, it is only the inclusion of randomisation which meant that this study required UK research ethics approval.

There are some weaknesses in the selection of BP as the primary endpoint; however these effects should be the same in each arm of the study. GPs will commonly check BP a second time if it is raised, but not if it is normal. There can consequently be a tendency for regression towards the mean in people with raised BP that is greater than in those with normal BP. This effect will need to be taken into account in the interpretation of the results. It is possible that people with raised BP will be under-detected.

A further problem with BP is that it tends to be recorded in primary care with marked end digit preference (EDP); *i.e*., a preference for recording a zero or five as the terminal digit [49]. EDP can make BP measurement a very blunt instrument, and make it harder to detect change. Although there has been improvement (*i.e*., a reduction) in EDP, especially in people with raised BP or cardiovascular co-morbidities, this remains a significant problem. Although, likely to influence each arm equally, EDP reduces the fidelity of our observations.

Routinely collected data are not like trial data; they are recorded inconsistently and reflect the primary healthcare professional's understanding of the problems presented. The record entries are made within the context of a short primary care consultation; what is recorded in the record is not a neutral act and often has connotations for patients (*e.g*., 'You told me my kidney blood tests are OK but you have labelled me as having CKD') [50]. We are only extracting coded data, and will not have access to free text data where other key data my lie. For example, 'urine NAD' (NAD = no abnormality detected) – a negative urine stick test may be recorded in the records; but as it has not been coded this test will remain hidden. Similarly, hospital letters and reports where the text has not been coded will also remain invisible to our searches.

Some members of the project team have been involved in the development of ABE as a quality improvement intervention for some time (SdeL, TC, JvV, NH) [19-21]. However, we have no personal stake that we feel will bias the outcome of this trial, and building-in independent scrutiny of the data should help ensure this is a fair test.

There are also a number of external pressures that are influencing the study; the most important are QOF CKD Indicator [51] and NICE guidance [4] issued in September 2008. The CKD QOF indicator is progressively being aligned with NICE guidance; and it is possible that these influences may be greater than any effect from the study. However, these are also factors which will equally influence all three arms of the study.

## Conclusion

This study should provide useful information about the influence of straightforward quality improvement interventions on the management of CKD; and if they are additive on the influences of financially incentivised QOF and the new national guidelines (NICE). The study will face all the challenges associated with working with routinely collected data, as well as the many confounding factors. We anticipate reporting whether the QI interventions tested have a place in improving the management of CKD.

## Competing interests

SdeL is the GP expert advisor for the QOF CKD Indicator. SdeL has received funding for research staff from Roche for the data analysis which formed part of the NEOERICA study (Refs: 7,9,18 and 36 are papers arising from this study). He has received sponsorship from Pfizer to speak at two cardiovascular meetings in 2008; received an honorarium for writing a magazine article (Presecriber) joint with HG. HG is a panel member expert advisor for the QOF and has received funding from several pharmaceutical companies for educational presentations on CKD, and an honorarium from a GP magazine to write an article on CKD (joint with SdeL). NT Funding: Grants Hospital Savings Association – £5,000 Kidney Research UK/British Renal Society – £45,000 Insulin Dependent Diabetes Trust – £7,000 SW Thames Kidney Fund – £10,000. Funding: others in last 5 years (for teaching and conference presentations) Baxter Healthcare, Roche, Novartis, Guys and St Thomas's NHS Trust, University of Warwick. JvV: For two years JvV's salary was part funded by the NEOERICA study (see SdeL) NJ Funding: Grants (DoH and BLF) ABLE – £92,182 Type 2 Diabetes – £248,155 Beliefs and attitudes to organ donation – £203,464 Ethnic differences in end of life care – £44,9141 Community ABLE toolkit – £20,000. NH received funding for MIQUEST query authoring as part of the NEOERICA study (see SdeL). KH Funding: Grants Pfizer International Doxazosin Award 2003: The role of alpha blockade on matrix synthesis by mesangial cells – £10,000 Pfizer award 2004: To investigate the effect of Atorvastatin on renal reperfusion injury – £12,000 Health Foundation 2007–2010: Quality Improvement in CKD: a challenge for primary care – £695,000 Edith Murphy Foundation 2007–2010: Quality Improvement in CKD due to diabetes – £450,000 LNR CLAHRC 2008–2014: Prevention of Chronic Disease and its Associated Co-Morbidity theme – c£4 million out of c£20 million total. Funding: others in last 5 years (travel support and ad hoc honararia) Roche, Ortho Biotech, Amgen, Baxter, Boehringher. Other: Advisory Board Membership Roche, Genzyme, Shire, Baxter, Novartis. MN, TC, AT, FR, EduB, IC: None declared.

## Appendix 1

Themes to be explored in the first year of the study

1. **Prevalence**. Prevalence of CKD, and prevalence by age band, for each of stage three to five CKD.

1.1 Practice prevalence (from serum creatinine records) compared with:

1.2 Population prevalence (from literature)

1.3 QOF prevalence (based on business case rules)

1.4 Standardised prevalence; deprivation and ethnicity recording

2. **Proteinuria recording**. Proportion of CKD patients with proteinuria estimation separately in diabetics and non-diabetics. Proportion of patients in whom proteinuria has been measured with albumin: creatinine ratio (ACR)>30 and >70 mg/mmol in non diabetics.

3. **BP**. Indicators of BP control.

3.1 Number of measurement in last 12 months

3.2 Most recent systolic and diastolic BP

3.3 Mean systolic and diastolic in last 6 months

3.4 Proportion meeting QOF standard, and NICE targets

4. **Angiotensin blockade in CKD**. Use of angiotensin modulating drugs (ACEI and ARB)

4.1 Total number of prescriptions

4.2 Use in CKD with proteinuria

4.3 Exemption coding

5. **Cardiovascular co-morbidities**. Prevalence, use of 10 year risk scoring.

6. **Process of delivering care**. Hints, tips, case-studies of how to achieve change (*e.g*., All hypertensives and those with CVS co-morbidity have a proteinuria test when having their blood tests.). Which primary care professionals are involved? Shift to primary care management.

7. **Motivation to change care**. Is CKD an illness? Are we inappropriately labelling much of the elderly population? Do the biomedical interventions do more good than harm?

8. **Improving the intervention**. How could the intervention be improved?

## Appendix 2

Themes for exploration in year two

1. **Programme fidelity and intervention exposure**. Has the implementation been feasible (programme fidelity) and what proportion of the practice have been interested in the feedback and results (intervention exposure)?

2. **How can the QI interventions be improved? **Suggested improvements to the interventions.

3. **Diabetes and CKD**. Prevalence of Diabetes and CKD and quality of management (comparing quality of management with QOF and national guidance, including new NICE guidance).

3.1 BP recording and control and use of angiotensin modulating drugs

3.2 HbA1c recording and value (compared with non-CKD diabetics, controlling for age and gender)

3.3 ACR (Albumin Creatinine ratio) in people with diabetes with CKD

3.4 Lipid management and use of statins and other medications for 1° and 2° prevention

3.5 Use of aspirin as primary and secondary prevention

4. **Cardiovascular co-morbidity**. We will look at risk factor management in people with cardiovascular disease, to include: use of lipid lowering therapy; use of aspirin; smoking cessation.

5. **Progression of CKD**. We will identify people with rapid progression.

6. **Anaemia and CKD**. We will flag people with anaemia who cross current NICE thresholds

7. **Avoiding harm**. We will look specifically for any evidence of increased numbers of falls; but are open to other unanticipated harmful consequences of the intervention.

8. **Good ideas**. The workshops will also seek to capture any examples of good practice and disseminate them across the group.

9. **Process of delivering care**. Any issues of call/recall of patients and concordance with therapy – especially angiotensin modulating drugs will be explored.

10. **Unexpected consequences**. We will try to identify any unexpected consequences of the interventions; good or bad.

## Appendix 3

Overview of the dataset extracted

Practice data

List size

QOF performance

Number and range of practice members engaged in CKD management

Pseudonymised practice indicator

Demographic

Age, gender

Ethnicity

Postcode (only first part is retained)

Index of deprivation (calculated in each practice from the postcode which is then deleted)

Cause of death & death

Clinical and laboratory

Serial measures of BP

Serial measures of serum creatinine concentration and eGFR

Co-morbid conditions (diabetes and its complications, ischaemic heart disease, heart failure, urinary obstruction)

Cardiovascular risk factors: smoking status; serum cholesterol and total cholesterol: HDL ratio; BMI, alcohol consumption; glycated haemoglobin and microalbuminuria in people with diabetes mellitus; urinalysis and total protein creatinine ratio; haemoglobin concentration

Lower urinary tract symptoms, prostate disease and urological factors which may reduce eGFR

Falls dataset (falls, likely fragility fractures, new diagnosis of osteoporosis)

Medications for optimal management that also impair renal function

Referral (to renal, diabetes, care of the elderly, urological and other specialties)

Other

Number of consultations in primary care

## Authors' contributions

SdeL conceived the original SGUL study and wrote much of the original St. George's application to the Health Foundation. He presented this at the funding meetings; he and MN created the combined bid which was funded by the Health Foundation. He is the principal investigator for the CRT. SdeL wrote the first draft of this paper with HG. HG worked closely with SdeL from the inception of the project and was a co-author in the original SGUL application to the Health Foundation. He is a senior investigator in the study protocol and co-wrote the first draft of this paper. TC has collaborated making many detailed contributions to the research protocol, and the developing study. TC has organised the SGUL study team. NT is one of the project co-ordinators for the study, responsible for recruiting and liaising with the southern locality practices. Contributed to the ideas behind the original grant proposal, attended the planning meetings, and helped edit the protocol also wrote parts of the organisational issues section. JvV designed the database and data management architecture for the study. MN worked with SdeL to create a single study from the originally separate bids. NJ: One of the project co-ordinators for the QI-CKD study, responsible for recruiting and liaising with the northern locality general practice. NJ also contributed to the development of the study. AT has generally contributed to the study through meetings and committees. He has also led on the development of a confidence questionnaire in general practice in managing chronic kidney Disease. EduB has contributed to the overall study and to the design of the economic evaluation. She has ensured that our dataset will be able to answer the research questions posed about cost effectiveness. IC has helped with the design of the in-depth process evaluation, the choice of focus groups, and the training of team members to run these. He will be responsible for the analysis of the data. NH has written all the MIQUEST queries used in the data collections for this study. He has also reviewed and contributed to the study design and methodology. FR worked with statistical colleagues to advise on the sample size and provided general specialist support for the development of this study. KH provided intellectual input to design of protocol, methodology, and execution.
